# Breast conserving treatment for breast cancer: dosimetric comparison of different non-invasive techniques for additional boost delivery

**DOI:** 10.1186/1748-717X-9-36

**Published:** 2014-01-27

**Authors:** Hilde Van Parijs, Truus Reynders, Karina Heuninckx, Dirk Verellen, Guy Storme, Mark De Ridder

**Affiliations:** 1 UZ Brussel, Vrije Universiteit Brussel (VUB), Brussels, Belgium

**Keywords:** Breast cancer, Tumor bed boost, Image guided radiation treatment (IGRT), Intensity modulated radiotherapy (IMRT), TomoTherapy, Vero

## Abstract

**Background:**

Today it is unclear which technique for delivery of an additional boost after whole breast radiotherapy for breast conserved patients should be state of the art. We present a dosimetric comparison of different non-invasive treatment techniques for additional boost delivery.

**Methods:**

For 10 different tumor bed localizations, 7 different non-invasive treatment plans were made. Dosimetric comparison of PTV-coverage and dose to organs at risk was performed.

**Results:**

The Vero system achieved an excellent PTV-coverage and at the same time could minimize the dose to the organs at risk with an average near-maximum-dose (D2) to the heart of 0.9 Gy and the average volume of ipsilateral lung receiving 5 Gy (V5) of 1.5%. The TomoTherapy modalities delivered an average D2 to the heart of 0.9 Gy for the rotational and of 2.3 Gy for the static modality and an average V5 to the ipsilateral lung of 7.3% and 2.9% respectively. A rotational technique offers an adequate conformity at the cost of more low dose spread and a larger build-up area. In most cases a 2-field technique showed acceptable PTV-coverage, but a bad conformity. Electrons often delivered a worse PTV-coverage than photons, with the planning requirements achieved only in 2 patients and with an average D2 to the heart of 2.8 Gy and an average V5 to the ipsilateral lung of 5.8%.

**Conclusions:**

We present advices which can be used as guidelines for the selection of the best individualized treatment.

## Background

Postoperative irradiation after breast conserving surgery (BCS) for breast cancer has shown a gain in recurrence free and overall survival [1-9]. An additional boost to the initial tumor bed has shown an additional gain in recurrence free survival [10]. In the last decades, a lot of attention has gone to the development of new techniques to reduce side effects. In case of breast irradiation this means late side effects on skin, heart and lungs. With this evolution, several techniques to deliver a boost dose to the initial tumor bed have become available. Historically the boost dose mainly was delivered by electrons. To date, it is unclear which technique should be preferred. A comparison of the boost techniques used in the EORTC 'boost versus no boost' trial showed no significant difference between electron, photon or interstitial boost in terms of fibrosis and local control [11,12]. But it was not the primary goal of this trial to investigate different outcome with different boost techniques. Differences within several photon boost techniques have not been investigated. It was our objective to make a dosimetric comparison of different non-invasive treatment techniques for additional boost delivery to offer an individualized best treatment to breast conserved patients.

## Methods

From a pool of available CT scans of early breast cancer patients treated in an earlier trial [13], 10 situations were selected. In this selection left and right breast cancer localizations were equally present. On each side a tumor located in each of the 4 different quadrants and a centrally located tumor was chosen. Within this selection, attention was given to select small as well as larger boost volumes, small as well as larger breast volumes and deeply as well as superficially located tumors.

A 'CTVboost' was drawn to include the site of the primary tumor, according to pre-operative imaging of the breast and according to the visual seroma and/or fibrosis on post-operative CT, with a margin of 7 mm in all directions to encompass potential microscopic disease extension. When present, surgical clips were to be within the CTVboost. The CTVboost excluded the skin, pectoralis muscle, ribs, lung and heart. PTVboost to CTVboost margin was 6 mm in all directions, but limited at the skin. The PTVboost, which could extend beyond the pectoralis major muscle/breast tissue interface, was used for determining the aperture of the treatment fields. A 'PTVboost-eval' was defined as the PTVboost limited at 5 mm below the skin surface. The PTVboost-eval was used for generating dose volume histograms (DVH) and comparative analyses. A margin of 5 mm was chosen to minimize the contribution of the dose build-up area at the skin. As organs at risk (OAR) the ipsilateral lung, heart, ipsilateral breast and contralateral breast were contoured.

Multiple treatment approaches were deployed for each CT set to deliver a dose of 16 Gy in 8 fractions of 2 Gy, which is the dose we prescribe in daily clinical practice in our department based on the EORTC boost versus no boost trial [12]. For each situation, all of following techniques were planned: electrons, a photon boost with 2 and 3 static fields and a photon boost with dynamic conformal arc using the CMS XIO planning software (Elekta AB, Stockholm, Sweden), a photon boost with the Vero^®^ system (joint product of BrainLAB; BrainLAB AG, Feldkirchen, Germany and MHI; Mitsubishi Heavy Industries, Tokyo, Japan) [14], which has the possibility to turn the ring-gantry from 30 to 330 degrees towards the table, a photon boost with the TomoTherapy^®^ system using rotational IMRT, as well as the static application (TomoDirect^®^) for tangential IMRT (Accuray Inc., Madison, USA) (Table 1).

**Table 1:** Used planning software

**Technique**	**Planning software**	**Type of calculation algorithm**[15]
Electrons	CMS XIO V4.64	b
Photons with 2 static fields	CMS XIO Release V4.62.00.13	b
Photons with 3 static fields	CMS XIO Release V4.62.00.13	b
Photons with dynamic arc	CMS XIO Release V4.62.00.13	b
Vero^®^	iPlan RT Dose 4.1.2 for Vero^®^	b
Photons with rotational IMRT (TomoTherapy^®^)	TomoTherapy Planning Station H-Art Version 4.0.5	b
Photon boost with tangential IMRT (TomoDirect^®^)	TomoTherapy Planning Station H-Art Version 4.0.5	b

Planning software per technique and corresponding type of calculation algorithm [15].

The planning aims were to cover 95% of the volume of the PTVboost with at least 95% of the prescribed dose, but not more than 107%. For all OAR, except for the ipsilateral breast, the volume receiving 5 Gy should not exceed 5%. For the ipsilateral breast there were no constraints, since today it is unclear which degree of dose spread within the breast should be considered as Acceptable. As an alternative to using constraints, several measurements for conformity were used. As a measure of low dose spread the ratio of the volume of the 20% isodose to the 95% isodose (Vol20/Vol95) and to the ratio of the volume of the 50% isodose to the 95% isodose (Vol50/Vol95) were registered. As a measure of conformity the conformity index (CI) was calculated, using the following formula [16]:

$$\mathrm{CI}=\;\frac{T{V_{\mathrm{PIV}}}^{2}}{\;\mathrm{TV}*\mathrm{PIV}\;}\;\begin{array}{l} \mathrm{TV}=\mathrm{target}\;\mathrm{volume}\\ \mathrm{PIV}=\mathrm{prescription}\;\mathrm{dose}\;\mathrm{volume}\\ TV_{\mathrm{PIV}}=\mathrm{overlap}\;\mathrm{of}\;\mathrm{TV}\;\mathrm{and}\;\mathrm{PIV} \end{array}$$

In this setting the TV is the PTVboost-eval, the PIV is the 95% isodose of 16 Gy.

**Figure 1:**
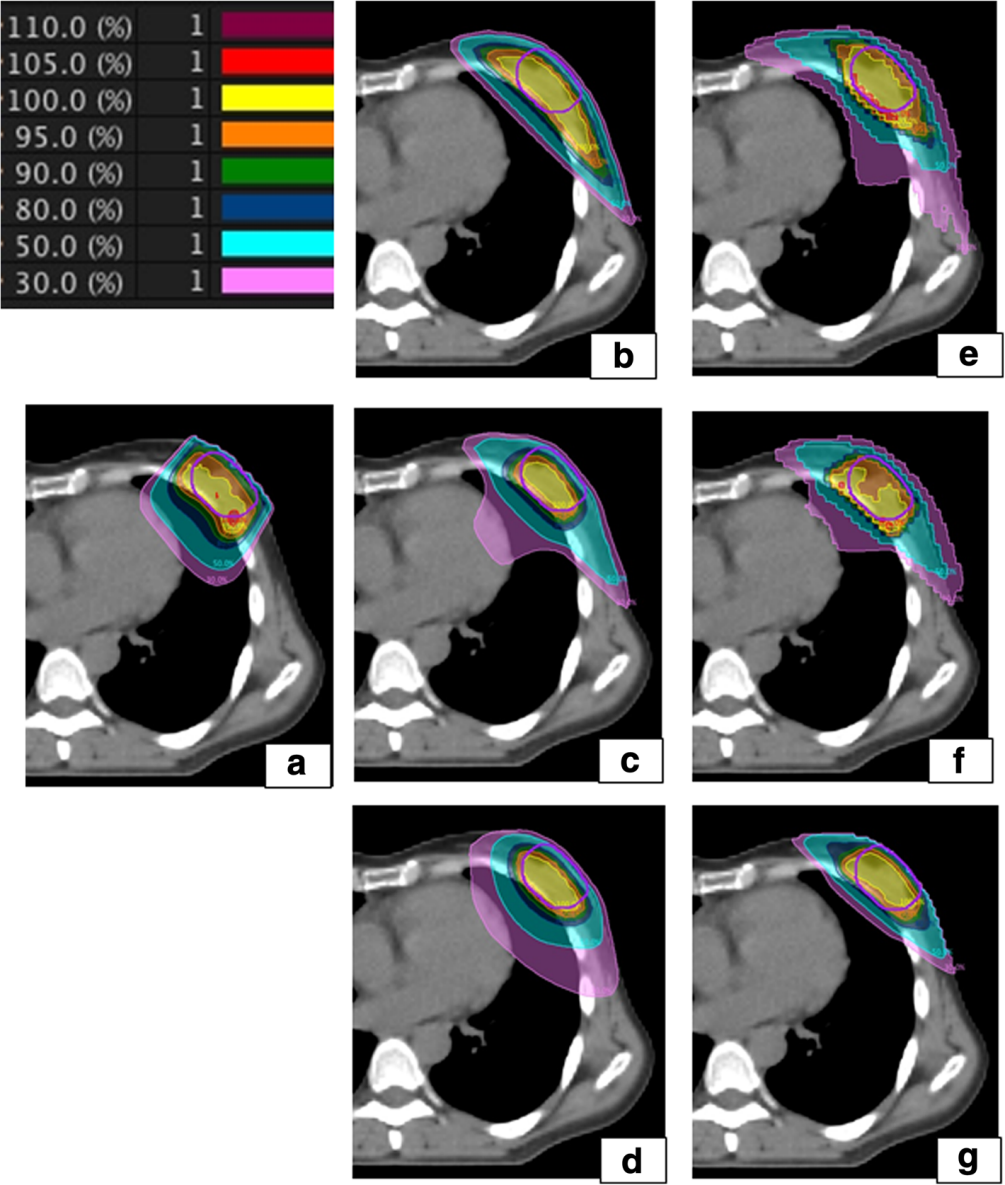
**Dose distribution for 1 patient for all techniques.** The dose distribution for 1 patient for all techniques: **(a)** electrons, **(b)** 2 tangential fields, **(c)** 3 fields, **(d)** arc, **(e)** Tomotherapy, **(f)** Tomodirect, **(g)** Vero.

Figure 1 shows the different planning techniques. For the electrons 1 beam perpendicular to the breast was used. The aperture of this beam was a rectangular shaped block surrounding the PTVboost to encompass it with the 95% isodose. The energy was chosen to reach the deepest point of the PTVboost with the 95% isodose. Available energies were in the range of 6 to 15 MeV. For the photon boost with 2 static fields either 2 tangential or 2 wedged fields could be used, depending on the localization of the PTVboost. When using the 3-field technique a perpendicular field to the breast was added to 2 tangential fields. For the planning on Vero 2 conformal tangential fields (ring 0°) were chosen to cover the PTVboost and avoid as much as possible the ipsilateral lung, heart and contralateral breast. Afterwards 2 more beams per tangential beam were added with the same gantry angle but different ring rotation (30° and 330°). As last part more conformal beams and compensation fields were added to reach a conformal dose distribution with a low dose to OAR. The maximum amount of beams per patient was kept at 10 to keep the treatment time acceptable. A treatment with 10 beams can take up to 25 minutes. TomoTherapy combines a rotational IMRT with a translational movement of the couch. Blocking structures and working volumes were used as was published earlier [17]. TomoDirect is the static application of TomoTherapy, where the gantry can be fixed at pre-chosen angles. Four tangential beams and 1 beam perpendicular to the breast were used to conform the dose.

## Results

Ten CT scans were selected for analysis. For both left and right side, a primary tumor location in each of the 4 quadrants was present, as well as a centrally located primary tumor bed. The pathological T-stage ranged from T1b to T2. The mean maximal diameter of the tumor was 1.6 cm (range: 0.6 - 2.7 cm). In 3 patients the deepest border of the tumor was located more than 3.5 cm from the skin surface. In only 1 patient the PTVboost did not reach the skin surface. The mean PTV volume was 71.73 cc (range: 24.91 - 137.88 cc). The mean volume of the ipsilateral breast was 447.92 cc (range: 108.66 - 865.74 cc). The mean PTV to ipsilateral breast ratio was 19.6% (range: 7.8% - 34.2%) with 2 patients having a ratio of more than 30%, 2 patients between 20 and 30%, 5 patients between 10 and 20% and 1 patient with a ratio of less than 10%. Not all 10 tumor localizations were clinically acceptable for electron boost delivery. In 2 patients the tumor was located too deep to cover the PTV with 15 MeV electrons. In the 3rd patient the tumor bed was located in the lateral breast fold. In practice, this patient would have been repositioned for electron boost delivery, which we could not do in this CT-based dosimetric comparison. For the goal of the comparison a dose distribution was calculated for all 10 CT sets, but for the interpretation of the further results, we should keep in mind that there are 3 irrelevant situations present.

The PTV coverage (95% of the PTV volume receiving 95% of the prescription dose) was achieved in 2 patients with electrons, in 7 with the 2-field photon boost, in 4 with the 3-field and the dynamic conformal arc photon boost and in all 10 patients with the rotational and static TomoTherapy modalities and Vero. The reason for failing to reach the PTV coverage criteria for the 2-field, 3-field and rotational photon boost, often was the build-up. The mean build-up was more than 5 mm for the 2-field, 3-field and dynamic conformal arc photon technique, was slightly less than 5 mm for Vero and electrons and was close to zero for the TomoTherapy modalities (Table 2). The CI was equal to or more than 60% in 2 patients with electrons, in none of the 2-field photon boost plans, in all 10 of the 3-field photon boost plans and Vero, in 9 of the dynamic arc plans, in 6 of the rotational and in 5 of the tangential TomoTherapy plans (Table 3). Today there is no literature available that gives an idea of which CI is acceptable. From this analysis, our conclusion is that a CI of 70% or more can be considered as excellent, between 60% and 70% as good, between 50% and 60% as acceptable and less than 50% as bad. The mean Vol50/Vol95 was more than 4 with the 3-field photon technique and with the tangential TomoTherapy technique, meaning that for these 2 modalities more than 4 times the volume of the actual target received an over-dosage of 50% of the prescribed dose or an over-dosage of 16% to the surrounding tissue, already treated to 50 Gy. Low dose spread was more present with dynamic arc photon boost and the TomoTherapy modalities, with a mean Vol20/Vol95 of more than 10. For the other techniques this ratio ranged between 5.8 and 9.6.

**Table 2:** Dose comparison

		**1**	**2**	**3**	**4**	**5**	**6**	**7**
**PTV**	**mean (sd) (Gy)**	16,17 (0,27)	16,13 (0,48)	16,02 (0,44)	15,98 (0,50)	16,07 (0,35)	16,17 (0,27)	16,07 (0,25)
	**V95% (%)**	79,91	94,44	94,51	93,15	96,70	97,58	97,90
	**CI (%)**	47 (2–63)	39 (21–57)	69 (60–84)	67 (54–73)	70 (64–78)	59 (50–66)	61 (54–82)
	**Vol50/Vol95**	3,62	2,90	4,91	3,86	3,22	3,62	4,42
	**Vol20/Vol95**	11,90	6,42	9,59	11,95	7,97	11,90	10,93
	**Mean build-up (mm)**	4.6	5.7	6.4	7.2	4.5	2.2	2.1
**Heart**	**mean (sd)**	0,36 (0,67)	0,05 (0,10)	0,47 (0,69)	0,80 (0,68)	0,23 (0,25)	0,15 (0,20)	0,54 (0,64)
	**D2 (Gy)**	2,75	0,33	2,48	2,68	0,94	0,85	2,26
	**V5 (%)**	1,20	0,00	0,27	0,43	0,00	0,02	0,01
**Ipsilat lung**	**mean (sd) (Gy)**	0,97 (1,97)	0,47 (1,41)	1,09 (1,85)	1,62 (1,98)	0,55 (1,01)	1,44 (1,78)	1,02 (1,46)
	**V5 (%)**	5,84	2,83	4,18	9,01	1,49	7,32	2,85
	**V8 (%)**	3,09	1,92	2,17	3,38	0,65	2,56	1,26
	**V15 (%)**	0,12	0,25	0,18	0,11	0,01	0,09	0,07
**Contralat breast**	**mean (sd) (Gy)**	0,00 (0,00)	0,02 (0,03)	0,01 (0,02)	0,31 (0,23)	0,05 (0,05)	0,04 (0,04)	0,05 (0,05)
	**D2 (Gy)**	0,00	0,10	0,07	0,81	0,25	0,15	0,20

Summary of dose comparison: (1) electrons, (2) 2 tangential fields, (3) 3 static fields, (4) arc, (5) Vero, (6) TomoTherapy, (7) TomoDirect; the build-up was evaluated in the 9 patients with PTV reaching the skin.

**Table 3:** **conformity index** (**CI**): **distribution per interval of 10**%

**CI**	**1**	**2**	**3**	**4**	**5**	**6**	**7**
**<50%**	4	9	0	0	0	0	0
**50–59%**	4	1	0	1	0	4	5
**60–69%**	2	0	5	5	5	6	4
**≥70%**	0	0	5	4	5	0	1

Conformity index for the different techniques, distribution per interval of 10%: (1) electrons, (2) 2 tangential fields, (3) 3 static fields, (4) arc, (5) Vero, (6) TomoTherapy, (7) TomoDirect.

The near-maximum-dose (D2) [18] to the heart for all techniques was below 5 Gy, except for 2 electron boost plans. One of these plans belonged to a patient who would, in good clinical practice, not have been a good candidate for electron boost delivery, because of the deep localization of the tumor bed. A rotational technique tends to deliver slightly more dose to the ipsilateral lung. Vero was the only technique which could keep the V5 for the ipsilateral lung below 5% in all cases. The arc technique with CMS and TomoTherapy delivered a mean V5 of 9% and 7% respectively, with electrons (not considering the 3 bad candidates) a mean V5 of 5% was delivered, for the other techniques the V5 ranged from 1.5 to 4% (Table 2).

All techniques were able to spare the contralateral breast with a near-maximum dose below 1.5 Gy. Only when using electrons, zero doses were delivered in all patients (Table 2).

## Discussion

We report a dosimetric comparison of different non-invasive techniques to deliver a boost dose after whole breast irradiation as part of the breast conserving treatment of breast cancer. We chose to select real cases for this comparison. The cases were part of a patient population of a phase III trial [13]. The target delineation was performed at the moment of the actual treatment planning according to the protocol of the trial and not as preparation for this work. This decreased the risk of unintentionally favoring a certain technique. With our selection we wanted to get a an idea of the different possibilities for a tumor localization in all of the four quadrants or a centrally located tumor on both sides. Within this selection we including small as well as larger breasts, small as well as larger target volumes and superficially as well as deeply located tumors.

A point of discussion could be the use of different planning software systems for the different techniques. Knöös reported a comparison of dose calculation algorithms [15]. Algorithms were divided into 2 groups: type a models, where changes in lateral transport of electrons are not modeled, and type b models, that in an approximate way consider changes in lateral electron transport. According to Knöös, the use of type b calculation algorithms will in general reduce the uncertainty in the delivered dose to the patient. For this analysis all planning software used type b calculation algorithms.

We concluded that Vero was the most promising technique, with the best median ranking (Table 3). The second best median ranking was seen for the photon boost with 2 fields. This was caused by a better sparing of OAR, but was at the cost of a worse PTV coverage and more dose spread to the already irradiated ipsilateral breast. Assuming that the PTV coverage should be the first concern, this "second place" is unjustified. Both TomoTherapy modalities were also promising, with a median ranking of 3 and 3.5. If paying even more attention to minimize the low dose spread, perhaps they could score even better. This is of course the difficult choice when using inverse planning systems. They are very dependent on the effort you are willing to invest. Once you achieve a clinical acceptable plan, do you accept it or do you try to achieve even better? The extra effort and time invested in further optimization of the dose distribution should be balanced to the expected clinical impact. In this work we compared only the boost dose of 16 Gy. Because, on itself, this is a low dose, less attention could have gone to the low dose spread when planning with TomoTherapy. The use of sophisticated IMRT-IGRT linear accelerators increases the cost of radiation treatment [19]. The expected clinical benefit should also be balanced to the financial aspect. On top of that, we should keep in mind that in clinical practice, a whole breast irradiation was already performed. Even a low dose on top of 50 Gy can be of clinical importance [20] (Table 4).

**Table 4:** Ranking

		**1**	**2**	**3**	**4**	**5**	**6**	**7**
**PTV**	**V95**	7	4	6	5	1	1	1
	**CI**	6	7	2	3	1	5	4
	**Vol50/Vol95**	3	1	7	5	2	3	6
	**Vol20/Vol95**	5	1	3	5	2	5	4
	**Build-up**	4	5	6	7	3	2	1
**Heart**	**D2**	7	1	4	6	3	2	4
	**V5**	7	1	5	6	1	1	1
**Ipsilat lung**	**V5**	5	2	4	7	1	6	2
	**V8**	6	3	4	7	1	5	2
**Contralat breast**	**D2**	1	3	2	7	6	4	5
**TOTAL SCORE**		51	28	43	58	21	34	30
**MEDIAN RANKING**		5.5	2.5	4	6	1.5	3.5	3

Ranking of the different techniques according to the dose comparison, techniques: (1) electrons, (2) 2 tangential fields, (3) 3 static fields, (4) arc, (5) Vero, (6) TomoTherapy, (7) TomoDirect; scoring from 1 to 7, with 1 being the best score; the build-up was evaluated in the 9 patients with PTV reaching the skin.

None of the used techniques seem significantly influenced by respiratory movements. Electrons probably are little affected, since they are delivered with an applicator touching the skin. Wedged techniques or field-in-field techniques seem insensitive for breathing motion, while IMRT techniques are highly sensitive to movement [21]. Tomotherapy uses IMRT, but plan delivery accuracy doesn't seem significantly affected by breathing [22].

The time per session for the patient on the treatment table differs a lot between the different techniques. Based on our clinical experience in our department, a treatment with electrons mostly takes less than 5 minutes. The delivery of a photon boost with 2 or 3 fields or arc with Elekta can take about 12 minutes. A treatment with Vero or Tomotherapy on average takes 20 minutes. The difference between the electron and photon treatments for a large part is caused by the cone beam and image registration, which is performed daily for all photon techniques.

Today, Vero and Tomotherapy proofed state of the art. If not available, the 3-field photon technique showed the best results. The use of an arc did not deliver a substantial benefit for the conformity, but did cause more low dose spread. Electrons delivered a worse PTV coverage and more doses to the ipsilateral lung and heart. However, there are a lot of things to be considered when interpreting these results. In our department, when we use electrons, we choose the energy to encompass the deepest border of the target volume with the 85% isodose. In this analysis, we wanted to encompass the target with the 95% isodose to be able to do a comparison with photons. In daily practice, we would not give such a high dose to the OAR as was shown in this work, but the PTV coverage would be even worse. Although electrons deliver a less optimal dose distribution and despite of the fact that electrons are used for boost delivery routinely, no differences in local recurrences are seen between electron and photon boost techniques. In the EORTC boost versus no boost trial [11] there was no statistically significant difference in 5-year local failure rates between electron and photon boost. Another consideration is the localization of the target. Electrons are not used in all situations. There were 2 patients with a tumor bed located too deep to be covered with electrons with energy of 15 MeV. In our department we would choose a photon boost in these cases. One tumor bed was located laterally, where the breast formed a fold. In practice we would have repositioned the breast to have a smooth surface, which could not be done on this CT-based planning analysis, which resulted in an irrelative dose distribution.

In 9 of the 10 patients, the PTV extended to the skin surface. When you want to deliver dose at the skin surface, it is important to choose a technique which has little build-up. We see that a rotational technique has a larger build-up area than the other techniques, except for TomoTherapy. In case it is clinically important to deliver dose at the skin surface, other techniques or the use of a bolus should be considered. When the target is located at the thoracic wall, rotational techniques tend to spread low dose to a larger volume of the ipsilateral lung and heart. In our choice for the most ideal technique, we should balance what is less damaging: a slightly higher dose to a smaller part of the OAR or a lower dose spread to a larger volume of the organ. For the heart, dose dependent regional cardiac perfusion defects are described [23]. Considering that part of the left ventricle already has received 50 Gy with the wide tangential fields, it would probably be better to minimize the volume of heart receiving extra dose by the boost delivery [20,24]. This means a technique that can avoid delivering dose in the direction of the left ventricle should be preferred. The same could be true for the ipsilateral lung. Verbanck et al. [25] showed that regional dysfunctions can be detected in the parts of the lung irradiated with the wide tangential fields. When using IMRT to minimize the dose to the lung, no changes were detected. For the boost delivery, theoretically, it is probably better to minimize the volume of ipsilateral lung receiving extra dose and thus minimize the volume at risk for damage. In practice, even with regional lung dysfunctions measured, there were no subjective implications on breathing or physical efforts in all patients.

We reported the CI. We chose a formula which considers both the over and under dosed areas [15]. However, we do not know the ideal CI to aim for, since there is no literature on this for breast irradiation. From this analysis, our own conclusion is that a CI of 70% or more can be considered as excellent, between 60% and 70% as good, between 50% and 60% as acceptable and less than 50% as bad (cfr Table 2). The used formula for CI does not give a view of the low dose spread. To have a more complete comparison of different techniques, you should analyze both the CI and some measure of low dose spread. We looked at the Vol20/Vol95 and to the Vol50/Vol95. The Vol20/Vol95 showed more differences for the different techniques, but the Vol50/Vol95 is perhaps of more clinical meaning.

From this work we derived advices which can be used as guidelines for selection of the best individualized boost technique. If available, Vero should be used, since it was the only technique that achieved an excellent PTV-coverage and at the same time could minimize the dose to the OAR. As a second choice, the TomoTherapy modalities proved to be a good alternative. Special attention should go to strict constraints to the OAR. A V5 < 2% to the heart and < 15% to the ipsilateral lung and a D2 < 1 Gy for the contralateral breast and < 3 Gy for the contralateral lung should be aimed at. If no IMRT-IGRT technique is available, the best choice depends on the localization of the tumor bed. In case of a superficial tumor bed, reaching the skin surface, one should avoid using a rotational technique, unless bolus is used. In case of a deep tumor bed reaching the thoracic wall, a 3-field technique is preferable. A slightly higher dose will be given to a smaller volume of the OAR, which is preferred above a rotational technique which spreads a lower dose to al larger volume. In case the tumor bed is surrounded by breast tissue, a conformal arc can be preferred above a 3-field technique. An arc technique delivers a better conformity, though there is more spread of very low dose to a larger volume of breast tissue. In most cases a 2-field technique shows an unacceptable conformity to be used in modern radiotherapy. Electrons should be reserved for a very superficially located tumor bed without contact with the thoracic wall. We strongly advice to avoid the use of an energy higher than 12 MeV.

## Conclusions

We performed a dosimetric comparison of different non-invasive techniques to deliver an additional boost after whole breast irradiation as part of the breast-conserving therapy. Guidelines were presented. Standard procedures should be replaced by an individualized treatment. The presented guidelines can help to find the best treatment technique for the individual patient.

## Abbreviations

BCS: Breast conserving surgery; CI: Conformity index; CT: Computer tomography; CTV: Clinical target volume; D2: Near-maximum-dose; DVH: Dose volume histogram; EORTC: European organization for research and treatment of cancer; Gy: Gray; IGRT: Image guided radiation treatment; IMRT: Intensity modulated radiotherapy; MeV: Mega electron volt; OAR: Organs at risk; PIV: Prescription dose volume; PTV: Planning target volume; TV: Target volume; TVPIV: Overlap of TV and PIV; V5: Volume receiving 5 Gy; Vol20/Vol95: Ratio of the volume of the 20% isodose to the 95% isodos.

## Competing interests

The UZ Brussel and Brainlab AG (Feldkrichen, Germany) have ongoing research collaboration.

## Authors’ contributions

HVP and TR were responsible for the conception and design of the trial. HVP, TR and KH were responsible for the acquisition of the plans and the dosimetric data. HVP and TR did the analyses, the interpretation of data and drafted the manuscript. DV, GS and MDR revised the manuscript and advised in the conception and design of the trial. All authors gave final approval of the version to be published.
